# The Metabolically Obese, Normal-Weight Phenotype in Young Rats Is Associated with Cognitive Impairment and Partially Preventable with Leptin Intake during Lactation

**DOI:** 10.3390/ijms25010228

**Published:** 2023-12-22

**Authors:** Margalida Cifre, Andreu Palou, Paula Oliver

**Affiliations:** 1 Nutrigenomics, Biomarkers and Risk Evaluation (NuBE) Group, University of the Balearic Islands (UIB), 07122 Palma, Spainandreu.palou@uib.es (A.P.); 2 CIBER of Physiopathology of Obesity and Nutrition (CIBEROBN), Instituto de Salud Carlos III, 28029 Madrid, Spain; 3 Health Research Institute of the Balearic Islands (IdISBa), 07120 Palma, Spain

**Keywords:** cognitive function, visceral obesity, perinatal programming, leptin, prevention, biomarker

## Abstract

The intake of high-fat diets (HFDs) and obesity are linked to cognitive impairment. Here, we aimed to investigate whether an early metabolically obese, normal-weight (MONW) phenotype, induced with an HFD in young rats, also leads to cognitive dysfunction and to evaluate the potential cognitive benefits of neonatal intake of leptin. To achieve this, Wistar rats orally received physiological doses of leptin or its vehicle during lactation, followed by 11 weeks of pair-feeding with an HFD or control diet post-weaning. Working memory was assessed using a T-maze, and gene expression in the hippocampus and peripheral blood mononuclear cells (PBMCs) was assessed with real-time RT-qPCR to identify cognition biomarkers. Young MONW-like rats showed hippocampal gene expression changes and decreased working memory. Animals receiving leptin during lactation presented similar gene expression changes but preserved working memory despite HFD intake, partly due to improved insulin sensitivity. Notably, PBMC *Syn1* expression appears as an accessible biomarker of cognitive health, reflecting both the detrimental effect of HFD intake at early ages despite the absence of obesity and the positive effects of neonatal leptin treatment on cognition. Thus, the MONW phenotype developed at a young age is linked to cognitive dysfunction, which is reflected at the transcriptomic level in PBMCs. Neonatal leptin intake can partly counteract this impaired cognition resulting from early HFD consumption.

## 1. Introduction

Individuals classified as metabolically obese but normal weight (MONW) represent a phenotype that, despite having a BMI within the normal range, exhibits metabolic features, including increased visceral adiposity, elevated levels of fasting glucose and triglycerides, insulin resistance, and hypertension, typically associated with obesity [1,2]. Unbalanced macronutrient diets, rich in fats or simple carbohydrates, contribute to the prevalence of the MONW phenotype [3,4,5], which accounts for approximately 20% of the global population [6]. This represents a significant public health concern. Unhealthy dietary patterns, obesity, and metabolic syndrome have been recognized as contributors to cognitive disorders [7,8]. Moreover, we demonstrated for the first time that not only obesity but the MONW phenotype, induced in adult Wistar rats with prolonged high-fat diet (HFD) consumption, is also linked to cognitive impairment [9]. Furthermore, we showed that peripheral blood mononuclear cells (PBMCs), increasingly used as a source of early disease biomarkers, can serve to identify transcriptomic biomarkers of cognitive decline. It has been described that PBMCs can reflect altered gene expression patterns occurring in the hippocampus of Alzheimer’s disease patients [10]. Similarly, we found that PBMCs can reflect early gene expression changes in the hippocampus of MONW-like rats compatible with impaired cognition [9].

Early life is a period of exceptional sensitivity to environmental factors, including diet [11,12,13]. Actually, perinatal nutrition and early life exposure to bioactive compounds can have lasting effects on adult health [14]. Different studies have shown that perinatal factors, like nutritional status, significantly impact the structure and function of the hippocampus [15], which, in turn, can affect adult cognitive function and susceptibility to brain diseases [16]. Breastfeeding, compared with formula feeding, is associated with a reduced risk of later obesity and related complications [17,18]. Maternal milk contains bioactive molecules, including leptin, which can play a role in perinatal programming [13,19]. Typically, leptin was heralded as a hormone mainly produced by adipose tissue, serving a crucial role as a signal for body adiposity. This protein acts within the hypothalamus to diminish food intake and regulate energy homeostasis [20]. However, high circulating leptin levels, such as those present in the obese state, can cause leptin resistance and are ineffective in regulating body weight [20]. In addition, the most recently identified function of leptin is as an essential nutrient during early postnatal development [13]. In newborns, a variety of nutrients and regulatory components are transmitted from the mother to the infant through breast milk. In 1997, Casabiell et al. confirmed the presence of leptin in both human and rat milk [21]. Their findings indicate that, in lactating rats, leptin is transferred through milk to the stomach and then enters the circulation of the infant rat in an intact form. Furthermore, our group demonstrated that leptin orally administered to neonate rats resists digestion, can be directly absorbed by the immature stomach, and regulates feeding [22]. Subsequently, we widely contributed to show that perinatal leptin intake can protect against obesity (reviewed in [13]), but also against the MONW phenotype in adult rats [23]. Leptin influences not only energy balance but also has a range of roles in the hippocampus and could be of special relevance during its development, acting as a cognitive enhancer [24]. Thus, we hypothesize that neonatal leptin, administered at physiological levels to avoid the potential development of leptin resistance at this critical period, could have a protective role against cognitive impairment induced by fat-rich diets.

Here, we aimed to investigate whether MONW-like rats, due to HFD consumption at early life stages (young adulthood), exhibit cognitive deficits described beforehand in adult rats and to reinforce early biomarkers of cognitive impairment previously identified in PBMCs. Furthermore, we assessed whether neonatal leptin intake could have potential beneficial effects in maintaining cognitive function in young rats exhibiting a MONW-like phenotype.

## 2. Results

### 2.1. Body Weight, Adiposity, and Serum Parameters

Animals treated with oral physiological doses of leptin did not differ in their body weight or fat mass content to control animals throughout the lactation period (data not presented). Key anthropometric and serum parameters of the three groups of this study (NF, HFD, and HFD-Lep) described in the Section 4 were previously published as part of a larger study aimed at assessing the impact of neonatal treatment with leptin, with the bioactive leptin sensitizing compound celastrol or their combination, on metabolic health [23]. In this previous manuscript, the effect on cognitive health was not addressed. The most relevant data are summarized in Table 1. As expected, after 11 weeks of dietary intervention, the HFD pair-feeding showed no impact on body weight. However, the HFD group exhibited a noteworthy increase in fat mass content and adiposity index and presented greater fat depots, including both subcutaneous (inguinal) and visceral (epididymal, mesenteric, and retroperitoneal) fat. Additionally, this group displayed higher levels of leptin compared with the control (NF) and leptin-treated animals (HFD-Lep), along with signs of insulin resistance, such as increased fasting glycemia and insulinemia and increased HOMA-IR index. Despite the Lep-HFD group having a higher adiposity index and visceral fat mass content compared with the control group, certain parameters remained unaffected even after prolonged exposure to a hyperlipidic diet in adulthood. Thus, rats supplemented with leptin during lactation maintained leptin levels and preserved features of insulin resistance, i.e., the HOMA index. Moreover, some of the fat depots (epididymal and mesenteric) were not increased in the Lep-HFD group, and their proportion of fat mass attained levels that fell between those observed in the control and HFD groups.

### 2.2. Effects on the Working Memory of a Young MONW Phenotype Induced with Isocaloric HFD Feeding in Animals That Received or Did Not Receive Leptin Supplementation during Lactation

**Figure 1:**
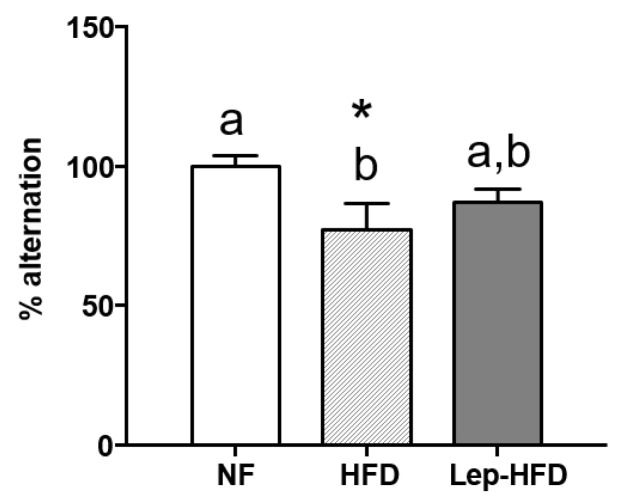
Impact of different diets on spatial working memory using spontaneous alternation paradigm in a T-maze. Groups are the same as those described in Table 1. Five trials per rat were performed, and the percentage of alternation was computed for each respective group. Results are expressed as mean ± SEM (*n* = 8 in the control NF and HFD groups; *n* = 10 in the Lep-HFD group). Statistical analysis: significant differences indicated by values with non-shared letters (a, b) (one-way ANOVA, *p* < 0.05). The LSD post hoc was used following the ANOVA analysis. Additionally, asterisks (*) indicate a significant difference compared with the control group (Student’s *t*-test, *p* < 0.05).

We assessed spatial working memory (short-term memory) using the spontaneous alternation paradigm in a T-maze. As shown in Figure 1, the HFD group demonstrated a significantly lower preference for alternation compared with the control animals fed a standard NF diet. Notably, animals that were orally supplemented with leptin during the lactation period preserved their performance in the T-maze, exhibiting outcomes comparable to the control group, despite being exposed to an HFD later in life.

### 2.3. Effects on Hippocampal Gene Expression of a Young MONW Phenotype Induced with Isocaloric HFD Feeding in Animals That Received or Did Not Receive Leptin Supplementation during Lactation

**Figure 2:**
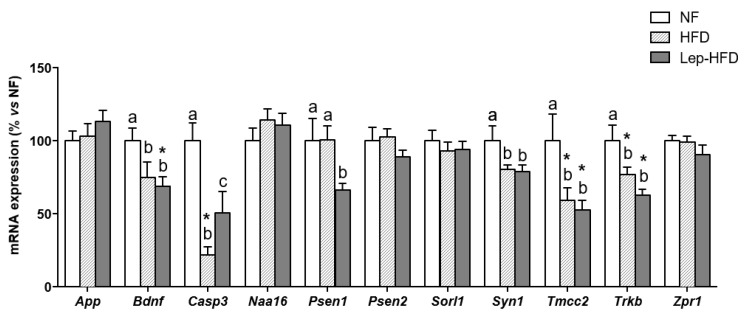
Impact of different diets on the expression of genes associated with cognitive impairment in the hippocampus. Groups are the same as those described in Table 1. Real-time RT-qPCR was used to quantify mRNA expression levels. Results are expressed as mean ± SEM (*n* = 8 in the NF and HFD groups; *n* = 10 in the Lep-HFD group) of the ratios of specific mRNA levels normalized against *Gdi1*, which served as a reference gene. The data for the control group were established as 100%, serving as a reference to the rest of the values. Statistical analysis: same as that described in Figure 1.

In the hippocampus (Figure 2), HFD pair-feeding led to adverse changes in the expression of selected cognitive-related genes. The genes analysed were chosen based on our previous expertise in identifying them as relevant biomarkers of cognitive impairment linked to the intake of fat-rich diets [9]. These genes participate in different, yet intertwined, processes: amyloid peptide metabolism (*App*, *Naat16*, *Psen1*, *Psen2*, *Sorl1*, and *Tmcc2*); neuronal viability/function (*Bdnf* and *Casp3*); synaptic function/signalling (*Syn1* and *Trkb*); and transcription factor-mediated regulation of cognitive-related processes (*Zpr1*).

In accordance with impaired cognitive function, a down-regulation in mRNA levels of genes encoding for the brain-derived neurotrophic factor (BDNF) and its receptor TrkB, along with the genes *Syn1* and *Tmcc2* following chronic exposure to the HFD, was observed. This trend persisted even in animals that were treated with leptin. Unexpectedly, the HFD also induced a notable reduction in the expression of *Casp3*, a gene encoding a proapoptotic protein. This decrease was partially mitigated in the Lep-HFD group. Interestingly, the Lep-HFD group exhibited reduced expression of *Psen1*, coding for a crucial protein linked to the metabolism of the amyloid precursor protein (APP) and associated with Alzheimer’s disease (AD). However, *Psen2* remained unaffected in all groups. In contrast to a previous study in which we administered an isocaloric HFD during adulthood and for an extended duration (16 vs. 11 weeks in the current study) [9], no changes in the expression of *App*, *Naa16*, *Sorl1*, and *Zpr1* genes were observed in the hippocampus of our young MONW-like animals.

### 2.4. Differential Expression of Genes Related to Cognitive Impairment in PBMCs of Rats with a Young MONW Phenotype Induced with Isocaloric HFD Feeding after Receiving or Not Receiving Leptin Supplementation during Lactation

**Figure 3:**
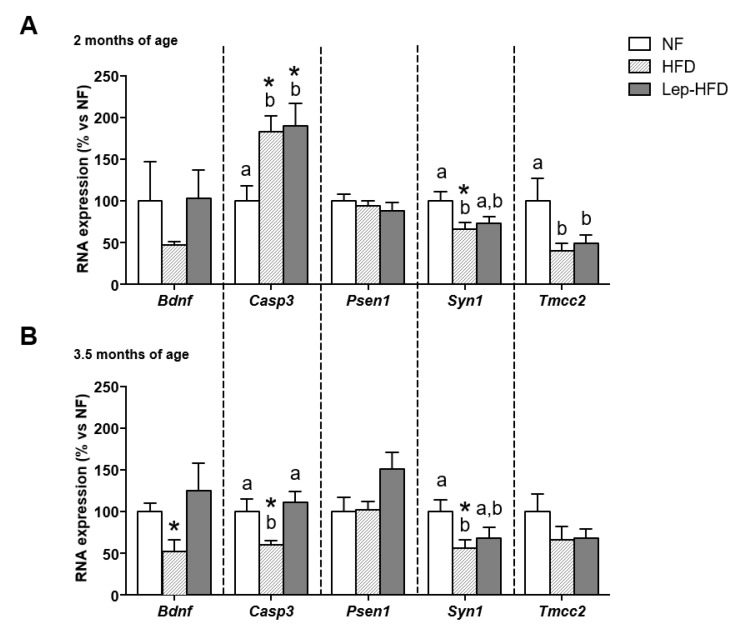
Impact of different diets on the expression of genes associated with cognitive impairment in PBMCs. Male Wistar rats of the groups explained in Table 1 at the age of 2 (**A**) and 3.5 (**B**) months. Real-time RT-qPCR was used to quantify mRNA expression levels. Results are expressed as mean ± SEM (*n* = 8 in the NF and HFD groups; *n* = 10 in the Lep-HFD group) of ratios of specific mRNA levels normalized against *Gdi1*, which served as a reference gene. The data for the control group were established as 100%, serving as a reference to the rest of the values. Statistical analysis: same as that described in Figure 1.

One of our aims was to assess whether PBMCs displayed comparable gene expression patterns to those observed in the hippocampus. To achieve this, we examined the mRNA levels of the most significant genes (those whose expression was altered in response to diet and leptin treatment in the hippocampus) in PBMC samples at 5 and 11 weeks of nutritional intervention (2 and 3.5 months of age, respectively). As shown in Figure 3, after just 5 weeks of HFD administration (2-month-old animals), we noted an upsurge in *Casp3* expression in both the HFD and Lep-HFD groups. This regulation was temporary, and by the conclusion of the experimental period, *Casp3* expression showed a decline in the HFD group but not in the Lep-HFD group, mirroring in PBMCs of leptin-treated animals the pattern observed in the hippocampus. Similarly, as observed in the hippocampus, there was a significant reduction in *Syn1* and *Tmcc2* expression in PBMCs after 5 weeks of exposure to the HFD. However, at the final time point (3.5-month-old animals), this decreased expression persisted only for *Syn1*. Interestingly, in contrast to the hippocampus, the reduction in *Syn1* mRNA levels in PBMCs was mitigated in the Lep-HFD group, presenting intermediate levels between the NF control and the HFD groups at both ages studied. Moreover, the HFD group exhibited decreased *Bdnf* mRNA levels in PBMCs, which became significant solely at the conclusion of the experimental period and was not observed in the Lep-HFD group, in contrast to the observations in the hippocampus. Notably, *Psen1*, which showed decreased expression in the hippocampus of the Lep-HFD group, remained unaltered in PBMCs. Lastly, we examined *Trkb* mRNA levels, given its altered gene expression in the hippocampus; however, it was found to be unexpressed in PBMCs.

### 2.5. Correlation Analysis

To gain further knowledge of the relationship between nutrition/adiposity and cognitive impairment, we performed a Pearson correlation analysis between gene expression and the anthropometric and serum parameters. The most outstanding finding was a negative correlation between *Syn1* mRNA expression in PBMCs and the HOMA-IR index (r = −0.562, *p* < 0.05), as well as with circulating glucose levels (r = −0.553, *p* < 0.05) when examining all the groups. It is worth mentioning that these correlations were not statistically significant in the hippocampus.

## 3. Discussion

While obesity had previously been related to cognitive impairment and dementia [8,25], our research was the first to demonstrate that the MONW phenotype induced in adult rats chronically fed with an isocaloric HFD is also linked to cognitive dysfunction [9]. Moreover, changes in key hippocampal gene expression were mirrored in PBMCs, highlighting their capacity as a source for transcriptomic biomarkers in cognitive research [9]. Here, we aimed to determine whether a MONW phenotype established with early-life consumption of an HFD immediately after the lactation period (not in adulthood) and for a shorter duration was also linked to cognitive impairment, as well as to analyse the likely preventive effect of perinatal leptin intake. The genes analysed in this study are the ones that were deemed noteworthy in the previously mentioned research.

Our results show that the expression of key genes involved in cognition was affected in the hippocampus in young animals with a MONW phenotype induced with the intake of an isocaloric HFD (60% kcal from fats) for 11 weeks (animals aged 3.5 months, compared with 6 months in our previous study). Specifically, we noted a decrease in the expression of *Bdnf* and its downstream effectors, including *Trkb* (encoding the BDNF receptor) and *Syn1* (coding for a protein essential for the release of neurotransmitters and synaptic function) [26,27]. Reduced expression of *Bdnf* is linked to reduced neuronal plasticity [28], which impacts processes involved in learning and memory. Notably, previous associations have been established between ad libitum HFD intake, obesity, and defectiveness in *Bdnf* expression [28]. Additionally, we observed a reduction in *Tmcc2* expression in the hippocampus, which is a gene whose disruption has been suggested as an indicator of AD [29]. These findings obtained in young MONW-like rats align with our earlier study involving older animals [9], suggesting that the dysregulation in the expression of these four genes (*Bdnf*, *Trkb*, *Syn1*, and *Tmcc2*) in the hippocampus, could serve as early indicators of deregulation linked to cognitive impairment. Our data also underline the dependence of hippocampal gene expression impairment on the duration of HFD exposure and the age of the exposed animals. We did not observe the same enhanced expression of *App*, indicative of AD-like pathology, that we described in adult animals subjected to the same isocaloric diet over a longer duration (16 weeks) [9] or described by other authors for animals maintained under ad libitum HFD [30]. Similarly, we did not confirm decreased expression of *Sorl1*, a gene associated with preventing amyloidogenic peptide production and deposition [31]. Similarly, we did not find the previously documented decrease in the expression of *Naa16*, whose encoded protein is known for inhibiting the secretion of amyloidogenic peptides [32]. In our current study involving young MONW-like animals, we identified a decrease in the expression of the proapoptotic marker *Casp3*, in contrast to our previous findings where adult MONW-like rats exhibited enhanced *Casp3* expression [9]. CASP3 has been linked to neurodegenerative processes and cell death, but recent research has suggested its role in modulating synaptic function [33]. The authors of the study suggested that the temporal activation of this protein is essential for synaptic function, with chronic activation being implicated in degenerative processes in the ageing brain. Hence, the distinct *Casp3* expression pattern observed across different lifetimes in our study could reflect its dual role in modulating synaptic function and apoptotic pathways.

There is a pressing need for early and accessible biomarkers for cognitive-related diseases, particularly considering that diagnoses for dementia and AD are typically confirmed when the condition is already manifest. Given the challenges associated with obtaining brain samples, the search for blood biomarkers of early cognitive impairment in the context of preventive medicine becomes relevant. PBMCs, comprising mainly lymphocytes and monocytes, are gaining prominence as a source of transcriptomic biomarkers due to their capacity to mirror gene expression changes occurring in internal tissues [34,35]. Our findings indicate that the expression patterns of the *Bdnf* and *Syn1* genes in PBMCs from young MONW-like animals (the HFD group) align with those observed in the hippocampus at the conclusion of the experiment (3.5 months of age). Furthermore, the expression of the *Syn1* gene in PBMCs, although not that of *Bdnf*, showed significant impairment (decreased expression) already earlier (2 months of age), suggesting its utility as an early marker for hippocampal alterations. In our previous study involving a MONW animal model with longer exposure to an HFD, we identified *Sorl1* and *Syn1* as potential indicators of early cognitive decline in PBMCs [9]. The utility of these biomarkers in PBMCs was subsequently validated by other researchers in humans with the MONW phenotype presenting mild cognitive impairment [36]. Therefore, considering our present results in both the hippocampus and PBMCs, the most robust finding is that obtained for *Syn1* gene expression. Reduced *Syn1* mRNA expression in the hippocampus has previously been linked to dysfunction at synaptic level [28]. The other two potential markers identified in the hippocampus, *Trkb* and *Tmcc2*, did not prove to be significant when analysed in PBMCs. *Tmcc2* initially mirrored hippocampal alterations in PBMCs after a month of nutritional treatment but lost significance afterwards. As for *Trkb*, it was not expressed in PBMCs at detectable levels.

The hippocampus is a brain region that mostly develops postnatally. Particularly, in rodents, the hippocampus develops between embryonic day 18 and postnatal weeks 2–3 [37]. Moreover, this brain region is highly plastic and rich in metabolic and stress hormone receptors [15]. Therefore, the hippocampus is greatly susceptible to the environment at the early stages of life. It is known that leptin receptors are expressed in many brain areas, including the hippocampus [38,39], suggesting a role for this protein in modulating neuronal function and enhancing cognition [40]. Specifically, leptin in the hippocampus plays a role in regulating neuronal function and survival and synaptic plasticity [41,42]. Leptin, a protein found in breast milk, could be especially relevant in neonatal periods, and it has been proposed to exert neurotrophic actions during the development of the hippocampus, acting as a cognitive enhancer [24,41,43]. However, there is scarce information available concerning the potential correlation between leptin supplementation during the suckling period and cognitive enhancements in later life. Our recent research demonstrates that administering leptin during the neonatal period can prevent some of the metabolic complications in animals with a MONW-phenotype induced with an HFD [23]. In that study, we did not address cognitive health. Here, animals that received physiological doses of leptin during lactation and were subsequently pair-fed with an HFD for 11 weeks exhibited the majority, though not all, of the molecular alterations observed in the hippocampus. This pattern was similar to that of rats that did not receive leptin but underwent the same hyperlipidic diet in adulthood. Remarkably, Lep-HFD rats appear to be less prone to cognitive impairment compared with their counterparts. This is supported by our behavioural findings. When we tested working memory using a T-maze spontaneous alternation paradigm, animals on an HFD exhibited impaired performance, consistent with transcriptomic changes observed in the hippocampus. However, rats that received leptin supplementation during lactation preserved their working memory even when exposed to an unhealthy dietary regimen in adulthood. Additionally, Lep-HFD animals exhibited diminished expression of *Psen1* in the hippocampus compared with both the control and HFD groups. PSEN1 plays a crucial role in beta-amyloid regulation and is closely linked to AD [44]. In fact, it has been suggested that inhibiting *Psen1* could serve as an approach for anti-amyloidogenic therapy in AD treatment [45].

Individuals with the MONW phenotype constitute a population vulnerable to the onset of metabolic syndrome, which encompasses insulin resistance among its associated conditions. In fact, HFD animals exhibited signs of insulin resistance. There is a well-established connection between AD and insulin resistance, with AD even being considered a new type of diabetes [46]. In contrast, leptin-treated animals showed an improved HOMA index despite being subjected to the same HFD. Then, a potential mechanism to contemplate, through which leptin might confer a positive impact on cognitive function in supplemented animals, is the enhancement in insulin sensitivity under an HFD. Indeed, it is recognized that reduced long-term potentiation (LTP), believed to underlie the formation of spatial memory, is linked with insulin resistance [47]. Leptin additionally supports synaptic plasticity in the hippocampus by enhancing LTP via responses mediated by NMDA receptors [48]. Administering leptin in early life may thus modulate NMDA receptor function and improve insulin sensitivity, which would facilitate working memory maintenance at later life stages. Remarkably, when analysing *Syn1* expression in PBMCs, but not in the hippocampus, animals treated with leptin under an HFD exhibited intermediate levels falling between those of the control and HFD groups, at both 2 and 3.5 months of age. This suggests that PBMCs may mirror transcriptomic changes before they become apparent in other tissues, such as the hippocampus. Hence, examining *Syn1* expression in PBMCs not only indicates the adverse effects of an HFD but also highlights the potential impact of neonatal leptin treatment in enhancing or preventing cognitive health. In fact, it is nowadays clear that leptin has a key action on hippocampal synapses, which is linked to pro-cognitive actions [41]. Moreover, we found an inverse correlation between PBMC *Syn1* expression and the HOMA-IR index, along with circulating glucose levels. Insulin resistance has been closely related to cognitive impairment progression [49]. Thus, impaired *Syn1* expression may reflect a higher cognitive risk due to increased insulin resistance. In line with this neuroprotective role of perinatal leptin, other researchers have recently demonstrated that cognitive impairment in neonatal rat offspring of diet-induced obese dams could be attributed to leptin withdrawal [50].

## 4. Materials and Methods

### 4.1. Animals and Experimental Design

The animal protocol was reviewed and approved by the Bioethical Committee of the University of the Balearic Islands, and university guidelines for the use and care of laboratory animals were followed. Two-month-old, virgin female Wistar rats were paired with male rats. These animals were procured from Charles River Laboratories (Barcelona, Spain). Following pairing, each female was individually housed with access to food and water. The rats were kept in a controlled environment with temperature at 22 °C and a 12-h light–dark cycle. Upon delivery, litters were standardized to contain 10 pups per dam. A total of 26 male pups were randomly divided into two groups: the control group (*n* = 16) and the leptin-treated group (*n* = 10). From day 1 to day 20 of lactation, during the first initial 2 h of the light cycle, the leptin-treated group received an oral solution of recombinant murine leptin (PeproTech, London, UK) dissolved in Captisol^®^. Conversely, the control group received Captisol^®^ (Ligand Pharmaceuticals, San Diego, CA, USA) in the same volume as the treated group. The leptin dosage was determined as five times the average daily leptin intake from mother’s milk, based on a prior study conducted by our group [22]. This leptin dose was considered as approaching physiological levels, considering the range of fluctuation in leptin levels found in nursing mothers’ milk. Captisol^®^ is a modified cyclodextrin designed to enhance the solubility, stability, and bioavailability of administered compounds through its unique chemical structure, by forming water-soluble complexes and avoiding crystallization [51]. As commented in the Section 2, this animal design is part of a larger study aimed at assessing the impact of neonatal treatment with leptin, with the bioactive leptin-sensitizing compound celastrol or their combination, on metabolic health (although the effect on cognitive health was not previously considered). Celastrol is a pentacyclic triterpenoid and for that reason, we used Captisol as a diluent to increase its solubility. Thus, though the celastrol group is not included in the present manuscript, and despite the recombinant leptin used being soluble in water, we used the same vehicle (Captisol) to allow group comparison in the original experimental design. Dilution of leptin in Captisol did not affect its role, since we obtained the same general metabolic programming protective effects as when administering leptin diluted in water [23].

On day 21, after weaning, both the control and leptin-treated male rats were individually housed and placed on a balanced control diet (D12450B, Research Diets). By day 23, the control rats were categorized based on body weight and randomly divided into two groups: the NF group (*n* = 8), which received a standard chow diet with 10% calories from fat (D12450B, Research Diets), and the HFD group (*n* = 8), which was fed with a chow diet containing 60% calories from fat (D12492, Research Diets). The sample size was determined based on similar experimental designs, indicating its adequacy for detecting statistical differences. To minimize potential confounding factors, both groups were matched by body weight. The leptin-treated group (*n* = 10) received the same hyperlipidic diet as the HFD group (Lep-HFD group). All diets were procured from Brogaarden (Gentofte, Denmark). These diets were administered for 11 weeks under isocaloric conditions relative to the control group, as previously detailed [52]. No blinding procedures were implemented.

At 14 weeks of age (3.5 months), the animals were euthanised under ad libitum feeding conditions. The hippocampus was promptly frozen in liquid nitrogen and stored at −80 °C for subsequent gene expression analysis. Different adipose tissue depots, including visceral and subcutaneous (epididymal, mesenteric, retroperitoneal, and inguinal) were removed and weighed to calculate the adiposity index.

### 4.2. Adiposity

Adiposity was assessed by calculating an adiposity index for each rat, obtained by summing the weights of epididymal, mesenteric, retroperitoneal, and inguinal white fat depots and expressing it as a percentage of total body weight. Additionally, fat mass was quantified using an EchoMRI-700™ (Echo Medical Systems, LLC., Houston, TX, USA) and expressed as a percentage of total body weight.

### 4.3. Blood Collection and PBMC Isolation

At the ages of 2 and 3.5 months, blood samples (1.5–2 mL) were obtained under feeding conditions from the saphena vein using EDTA 100 mM as an anticoagulant. The PBMC fraction was isolated using OptiPrep™ (Sigma Aldrich Química SA, Madrid, Spain) density gradient separation according to the manufacturer’s instructions. We used OptiPrep™ as the density gradient medium since it presents an optimum density for the isolation of rodent PBMCs from whole blood. Simultaneously, blood samples from the saphena vein were harvested without an anticoagulant, stored for 1 h at room temperature, and then centrifuged at 1000× *g* for 10 min at 4 °C to obtain serum samples. Additionally, animals underwent a 12–14 h nocturnal fasting period for serum collection to determine the HOMA-IR index using the formula by Matthews et al. [53].

### 4.4. Quantification of Circulating Insulin, Leptin, and Glucose Levels

Enzyme-linked immunosorbent assay kits were used to quantify insulin (Mercodia AB, Uppsala, Sweden) and leptin (R&D Systems, Minneapolis, MN, USA) levels in serum samples. Blood glucose was assessed using an Accu-Chek Glucometer (Roche Diagnostics, Barcelona, Spain). Both glucose and insulin levels were measured under fasting conditions.

### 4.5. Total RNA Isolation

Total RNA from both the hippocampus and PBMC samples was extracted using Tripure reagent (Roche Diagnostics, Barcelona, Spain) according to the manufacturer’s instructions. The RNA yield was quantified using a NanoDrop ND 1000 spectrophotometer (NanoDrop Technologies, Wilmington, DE, USA), and RNA integrity and purity were validated with 1% agarose gel electrophoresis. All samples used in this study presented 260/280 ratios ranging from 1.9 and 2.1, which is indicative of their purity and quality.

### 4.6. Real-Time Reverse Transcriptase Polymerase Chain Reaction (RT-qPCR) Analysis

For the hippocampus, 0.1 µg of total RNA (in a final volume of 5 µL) underwent denaturation at 65 °C for 10 min. Subsequently, reverse transcription to cDNA was performed using MuLV reverse transcriptase (Applied Biosystem, Madrid, Spain) at 20 °C for 15 min, 42 °C for 30 min, and a final step of 5 min at 95 °C. For PBMCs, equal amounts of total RNA (0.05 µg) were reverse transcribed into cDNA using an iScript™ cDNA synthesis kit (Bio-Rad Laboratories, Madrid, Spain). Both reactions were conducted in an Applied Biosystems 2720 Thermal Cycler (Applied Biosystem, Madrid, Spain). Following cDNA synthesis, real-time RT-qPCR was conducted to determine the mRNA expression of key cognitive impairment-related genes. As commented in detail in the Section 2, these genes were selected based on our previous expertise in identifying them as relevant biomarkers of cognitive impairment linked to the intake of fat-rich diets [9]. Each PCR was carried out using a diluted (1/10 for hippocampus and 1/5 for PBMC) cDNA template, forward and reverse primers (5 µM), and Power Sybr Green PCR Master Mix (Applied Biosystems, Madrid, Spain) following previously described conditions [54]. The threshold cycle (Ct) was calculated using the instrument’s software (StepOne Software v2.0, from Applied Biosystems, Madrid, Spain), and the relative expression of each mRNA was calculated as a percentage of control rats using the 2^−ΔΔCt^ method [55]. Data from the hippocampus and PBMCs were normalized against *Gdi1*. Primers for different genes were designed using the NCBI’s Primer-BLAST designing tool and are described in Table S1. *Gdi1* was selected as it has been previously identified as a reliable constitutive gene based on microarray studies [56]. All primers were procured from Sigma Genosys (Sigma Aldrich Química SA, Madrid, Spain).

### 4.7. Behavioural Testing: T-Maze Alternation

To evaluate the impact of dietary interventions on cognitive function, we used a spontaneous alternation paradigm in a T-maze, following the protocol from Deacon and Rawlins [57]. This is a widely used method in cognitive and behavioural neuroscience, particularly for rodents, recognized for its reliability, reproducibility, and effectiveness. It gives the possibility to analyse spatial working memory (short-term memory) based on the spontaneous behaviour of the animal without the need for incentives, punishments, or prior training, which is less stressful [58]. However, despite its usefulness for cognitive assessment, the T-maze test also has its limitations, e.g., since it is based on spontaneous behaviour, the effectiveness of the procedure depends on the animals’ motivation [58]. Also, the test focuses mainly on spatial memory and thus leaves other cognitive domains less explored.

At 3.5 months of age, rats underwent testing using a T-maze free-trial procedure. In summary, each rat was positioned at the start area of the T-maze and allowed to make a choice of which arm to enter. Once the rat made a selection, it was confined to that arm for 30 s, then returned to the start arm and confined there for 10 s. Subsequently, the animal had the opportunity to choose an arm again. This process was repeated for five trials per rat. A score of 0 was assigned if the rat chose the same arm, and a score of 1 was given if the animal alternated arms. A corrected percentage of success per animal was then calculated. Additionally, the latency (time taken by the animals to select the arm) was recorded to identify any potential delays in response, thus helping to rule out possible brain dysfunction that could impair decision processes.

### 4.8. Statistical Analysis

The data are expressed as means ± SEM. Normal distribution and homogeneity of variances were assessed using the Shapiro–Wilk test and the Levene test, respectively. Statistical significance was determined using an unpaired *t*-test to compare the HFD and HFD leptin-treated groups with the control group. Multiple comparisons were conducted using a one-way ANOVA followed by an LSD post hoc test. Log-transformed variables were used for statistical analysis when they did not meet parametric criteria. In the cases where a normal distribution or homogeneity of variances was not observed, nonparametric tests were applied. Linear relationships between key variables were evaluated using Pearson correlation coefficients. The statistical analyses were carried out using SPSS 28.0.0 for Windows (SPSS, Chicago, IL, USA), considering significance at *p* < 0.05.

## 5. Conclusions

Our preclinical study highlights the risks of unhealthy dietary habits during early life, which not only can lead to obesity but can also have significant consequences for cognitive health. We established a link between a young MONW phenotype, induced with exposure to a hyperlipidic diet, and cognitive dysfunction in male Wistar rats, even though this unhealthy diet was administered for a relatively short period. Furthermore, we propose that *Syn1* expression in PBMCs could serve as a readily detectable and robust early biomarker for cognitive impairment linked to the consumption of fat-rich unbalanced diets, though human validation is needed. Remarkably, we report the role of leptin intake during lactation inducing gene expression changes that could preserve cognitive function (as evidenced by preserved working memory) in MONW-like animals during adulthood. All in all, our results point to the importance of proper nutrition (including breastfeeding and balanced diets) during early life to preserve insulin sensitivity and protect not only against metabolic syndrome but also cognitive impairment. Our further research will be aimed at performing global gene expression analysis in samples from the hippocampus and PBMCs of MONW-like animals to gain knowledge on the mechanisms involved in altered cognition due to dietary unbalance, as well as to identify new early biomarkers of cognitive impairment. Furthermore, we aim to validate biomarkers obtained in PBMC, in rodents and in samples from human volunteers with MONW, overweight/obesity or different degrees of impaired cognition (from mild cognitive impairment to dementia).

## Figures and Tables

**Table 1:** Anthropometric and serum parameters.

	NF	HFD	Lep-HFD
Body weight (g)	409 ± 13	416 ± 6	410 ± 6
Fat mass (%)	19.4 ± 0.5 ^a^	23.9 ± 1.8 ^b,^*	22.9 ± 1.2 ^a,b,^*
Adiposity index (%)	8.36 ± 0.26 ^a^	11.2 ± 0.6 ^b,^*	10.4 ± 0.4 ^b,^*
Weight of adipose tissues (g)			
Epidiymal fat	9.59 ± 0.66 ^a^	13.7 ± 0.5 ^b,^*	11.1 ± 0.5 ^a^
Mesenteric fat	4.04 ± 0.26 ^a^	5.46 ± 0.4 ^b,^*	4.83 ± 0.30 ^a^
Retroperitoneal fat	11.0 ± 0.9 ^a^	13.1 ± 0.4 ^b^	13.2 ± 0.7 ^b^
Inguinal fat	9.73 ± 0.86 ^a^	13.9 ± 1.2 ^b,^*	13.5 ± 0.9 ^b,^*
Visceral fat content (g)	24.6 ± 1.5 ^a^	32.3 ± 1.0 ^b,^*	29.1 ± 1.2 ^b,*^
Leptin (ng·ml^−1^)—feeding	12.6 ± 1 ^a^	18.1 ± 2.3 ^b,^*	12.5 ± 1.3 ^a^
Glucose (mg·dl^−1^)—fasting	101 ± 3 ^a^	120 ± 7 ^b,^*	114 ± 4 ^a,^*
Insulin (µg·L^−1^)—fasting	0.18 ± 0.02 ^a^	0.33 ± 0.05 ^b,^*	0.24 ± 0.03 ^a,b^
HOMA-IR	1.32 ± 0.30 ^a^	2.5 ± 0.5 ^b^	1.7 ± 0.2 ^a^

The data pertain to male Wistar rats that were provided either a normal-fat (NF) control diet or a high-fat diet (HFD and Lep-HFD groups) from day 23 of age until they reached 3.5 months old. The HFD groups received isocaloric amounts of food compared with the control group. Visceral fat content: sum of the epididymal, mesenteric, and retroperitoneal fat depots. Results are expressed as mean ± standard error of the mean (SEM) (*n* = 8 in the control and HF groups; *n* = 10 in the Lep-HF group). Statistical analysis: significant differences indicated by values with non-shared letters (a, b) (one-way ANOVA, *p* < 0.05). The LSD post hoc was used following the ANOVA analysis. The absence of letters indicates no statistical difference. Additionally, asterisks (*) indicate a significant difference compared with the control group (Student’s *t*-test, *p* < 0.05).

## Data Availability

Data will be shared upon reasonable request to the corresponding author.

## Supplementary Materials

The following supporting information can be downloaded at: https://www.mdpi.com/article/10.3390/ijms25010228/s1.
