# Supplementary material for: The Metabolically Obese, Normal-Weight Phenotype in Young Rats Is Associated with Cognitive Impairment and Partially Preventable with Leptin Intake during Lactation
Source: Int J Mol Sci. 2023 Dec 22;25(1):228. doi: 10.3390/ijms25010228 (PMC10778589; doi:10.3390/ijms25010228)
Supplement: Supplementary file 1 [file ijms-25-00228-s001.zip › ijms-2745803-supplementary.pdf]

Table S1. Nucleotide sequences of primers used for real-time RT-qPCR amplification of cognitive impairment-related genes.

Gene	Forward primer (5'-3')	Reverse primer (3'-5')	Amplicon size (bp)
App	CTGCTGACCGAGGACTGAC	CCGAACTCCGCATCCATCTT	94
Bdnf	ATTAGCGAGTGGGTCACAGC	CGAGTTCCAGTGCCTTTTGT	189
Casp3	ACCCTGAAATGGGCTTGTGT	ACAGGTCCGTTCGTTCCAAA	280
Naa16	AGTGCTACCGAAATGCCCTCA	TGTGTCGGGCGTAACTGAAGA	139
Psen1	TGGTGTGGTCGGGATGATTG	CGACCAGCATACGAAGTGGA	203
Psen2	CCTGGCGGTGATGGAATACA	AGCGCTTCCCACAGTATGAC	292
Sorl1	CACCGTCTCATTGTCAGCAC	ATCTCGTAGCCCCTGGTTTC	123
Syn1	GCAGTTTGGTCATTGGGCTG	ACAGGGTATGTTGTGCTGCT	202
Tmcc2	TCCTCCTCTACCACCGATACC	CTCCCTTGTCACCCTTGTCC	103
Trkb	TCGGTATCACCAACAGCCAG	GCTCGGGGCAGAGGTTATAG	141
Zpr1	GGATTACCCTCCACATCACAG	TGTCTTTCAGCAGTCCTTCG	153
Reference gene			
Gdi	CCGCACAAGGCAAATACATC	GACTCTCTGAACCGTCATCAA	159

Abbreviations: *App*, amyloid precursor protein; *Bdnf*, brain derived neurotrophic factor; *Casp3*, caspase 3; *Naa16*, N(alpha)-acetyltransferase 16, NatA auxiliary subunit; *Psen1*, presenilin 1; *Psen2*, presenilin 2; *Sorl1*, sortilin related receptor 1; *Syn1*, Synapsin I; *Tmcc2*, transmembrane and coiled-coil domain family 2; *Trkb*, neurotrophic receptor tyrosine kinase 2; *Zpr1*, zinc finger protein 259.
